# Genome-wide characterization and evolutionary analysis of the *AP2/ERF* gene family in lettuce (*Lactuca sativa*)

**DOI:** 10.1038/s41598-023-49245-4

**Published:** 2023-12-11

**Authors:** Sunchung Park, Ainong Shi, Lyndel W. Meinhardt, Beiquan Mou

**Affiliations:** 1 US Department of Agriculture, Agricultural Research Service, Beltsville, MD 20705 USA; 2 Horticulture Department, University of Arkansas, Fayetteville, AR 72701 USA; 3 US Department of Agriculture, Agricultural Research Service, Salinas, CA 93905 USA

**Keywords:** Plant sciences, Plant stress responses, Abiotic, Molecular evolution

## Abstract

The APETALA2/ETHYLENE RESPONSIVE FACTOR (AP2/ERF) gene family plays vital roles in plants, serving as a key regulator in responses to abiotic stresses. Despite its significance, a comprehensive understanding of this family in lettuce remains incomplete. In this study, we performed a genome-wide search for the *AP2/ERF* family in lettuce and identified a total of 224 members. The duplication patterns provided evidence that both tandem and segmental duplications contributed to the expansion of this family. Ka/Ks ratio analysis demonstrated that, following duplication events, the genes have been subjected to purifying selection pressure, leading to selective constraints on their protein sequence. This selective pressure provides a dosage benefit against stresses in plants. Additionally, a transcriptome analysis indicated that some duplicated genes gained novel functions, emphasizing the contribution of both dosage effect and functional divergence to the family functionalities. Furthermore, an orthologous relationship study showed that 60% of genes descended from a common ancestor of Rosid and Asterid lineages, 28% from the Asterid ancestor, and 12% evolved in the lettuce lineage, suggesting lineage-specific roles in adaptive evolution. These results provide valuable insights into the evolutionary mechanisms of the *AP2/ERF* gene family in lettuce, with implications for enhancing abiotic stress tolerance, ultimately contributing to the genetic improvement of lettuce crop production.

## Introduction

Plants, as sessile organisms, constantly face challenges from various environmental stresses, such as cold, heat, drought, and salinity. These stresses can severely impact growth and productivity, leading to substantial reduction in crop yields^1,2^. Moreover, it has been projected that the increasing frequency of devastating heat, droughts, and other weather extremes due to climate change will cause around a 20% yield decline in major crops worldwide^3,4^. To cope with these challenges, plants have evolved complex regulatory mechanisms that enable them to respond and adapt to changing environmental conditions, while maintaining a balance between optimal growth and stress tolerance^5,6^. Key players in the regulatory mechanisms are transcription factors, which orchestrate plant responses to stresses by regulating the expression of various stress-responsive genes and modulating phytohormone signaling pathways^5,7,8^. Among these transcription factors, the APETALA2/ETHYLENE RESPONSIVE FACTOR (AP2/ERF) superfamily plays prominent roles in regulating various abiotic stress responses in plants^9–11^. The defining feature of the AP2/ERF superfamily is the presence of at least one AP2 domain^12,13^. The AP2 domain encodes a conserved DNA-binding sequence consisting of 60–70 amino acids, allowing the AP2/ERF proteins to directly interact with *cis*-acting elements, namely GCC box and/or C-repeat element (CRT)/dehydration responsive element (DRE), located in the promoter regions of downstream target genes^14–16^.

The AP2/ERF superfamily is categorized into four major subfamilies based on the number of AP2 domains, the presence of a B3 DNA-binding domain^17^, and sequence similarity: AP2, RELATED TO ABSCISIC ACID INSENSITIVE 3/VIVIPAROUS 1 (RAV), DEHYDRAION-RESPONSIVE ELEMENT BINDING FACTOR (DREB), and ERF subfamily^12,13^. According to the first comprehensive study of the AP2/ERF family in *Arabidopsis* and rice (*Oryza sativa*), the DREB subfamily is further divided into several subgroups, denoted as I–IV, while the ERF subfamily is divided into subgroups V–X^12^.

The function of *AP2/ERF* family genes in abiotic stresses has been extensively investigated in *Arabidopsis*^16,18,19^. Specifically, the *DREB* subfamily genes are well-known for their roles in abiotic stress responses, including cold, drought, and high salt conditions^16,20^. For example, in *Arabidopsis*, *C-REPEAT BINDING FACTOR* (*CBF*) genes—also known as *DREB1*—are rapidly induced by low temperatures and subsequently activate around 100 downstream target genes through interaction with CRT/DRE *cis*-elements in target promoters. These target genes collectively contribute to increased cold acclimation and freezing tolerance^5,8^. On the other hand, *Arabidopsis DREB2* genes are induced by drought and high salinity and activate genes involved in drought and heat tolerance, such as *LEAs* and heat chaperons, through binding to the same CRT/DRE *cis*-element^13,21^. The ERF subfamily, which primarily bind to ETHYLENE-RESPONSE ELEMENT (ERE) *cis*-elements with GCC-box sequences^11,22^, is generally considered to mediate ethylene-related responses. However, it also includes members that contribute to abiotic stress responses. For example, *Arabidopsis CYTOKININ RESPONSE FACTORS* (*CRFs*) such as *AtCRF6* and *AtCRF4* are induced by multiple abiotic stresses and positively regulate osmotic and freezing tolerance^23,24^. Other ERF subfamily members such as AtERF4^25^, AtERF7^26^, AtERF15^27^, and AtERF111^28^ play roles in abscisic acid signaling and are involved in responses to high salinity, osmolarity and hypoxia.

Lettuce *(Lactuca sativa*) is one of the most consumed vegetables globally^29^. It is a member of the Asterid clade and Asteraceae family with a chromosome number of 2n = 18^30^. Lettuce is recognized for its nutritional value and health benefits, serving as a rich source of essential vitamins, minerals, antioxidants, and dietary fibers^29^. Due to its herbaceous nature, lettuce is susceptible to various environmental stresses, including cold, heat, drought, and salinity^31,32^. These abiotic stresses can significantly impact lettuce growth, quality, and productivity, ultimately leading to yield loss^33,34^. Therefore, understanding the characteristics and evolutionary dynamics of the *AP2/ERF* gene family in lettuce is important for enhancing our knowledge of stress response mechanisms and developing strategies to improve lettuce crop resilience and productivity, especially in the face of climate change. A recent study characterized fourteen *CBF* genes, involved in freezing tolerance in lettuce, which are members of AP2/ERF family^35^. However, a comprehensive investigation into the entire AP2/ERF family in lettuce is still limited.

This study aimed to identify and characterize the AP2/ERF transcription factors in lettuce. Through an extensive search on the reference genome that is constructed from the cultivar ‘Salinas’^36^, we successfully identified a total of 224 *AP2/ERF* genes in lettuce. We investigated their genomic locations, exon–intron structures, and evolutionary relationships. The *AP2/ERF* genes were categorized into four distinct subgroups based on the number of AP2 domains, sequence similarity, and phylogenetic relationships. To gain insights into their functional roles, we assessed the expression of the *AP2/ERF* genes under various stress conditions, such as heat, cold, salt, and drought. Several *AP2/ERF* genes exhibited significant responses to all these stresses, making them promising candidates for further investigation and potential utilization in enhancing stress response in lettuce. We also explored gene orthology and duplication events within the AP2/ERF family to understand the genetic mechanisms that contribute to the expansion and functional diversification of this gene family in lettuce. Overall, this study contributes to our understanding of the evolutionary dynamics of the AP2/ERF transcription factor family and offers potential molecular targets for improving stress responses in lettuce.

## Results

### Identification of the AP2/ERF transcription factors in lettuce genome

To identify *AP2/ERF* family genes in lettuce, we queried the lettuce genomic protein database (version 8) using the Pfam model (PF00847) of the AP2 domain. This search led us to discover 223 genes that showed a significant match with the AP2 domain, all with an E-value of < 1e − 5. Previously, fourteen *LsCBF* genes in lettuce, belonging to the *AP2/ERF* family, were identified through a comparative phylogenetic analysis^35^, and the authors discovered that one gene (*Ls9g54101.1*) had been erroneously annotated as a splice variant in the genome, even though it encoded a distinct protein. Our analysis successfully identified all *LsCBF* genes except for the misannotated gene, and we included this gene manually in our analyses, bringing the total number of *AP2/ERF* genes in lettuce to 224 (Table S1). Among the 224 genes, twenty-four genes had two AP2 domains, 197 genes contained a single AP2 domain, and the remaining three genes had both AP2 and B3 domains.

The 224 *AP2/ERF* genes constituted approximately 0.59% of the total 37,826 coding genes in the lettuce genome. To compare this proportion with other species, we applied the same method to identify *AP2/ERF* genes in ten additional species, selected from the Asterid and Rosid clades, the two largest clades among flowering plants, with five species selected from each clade^37^ (Table S2). The percentages of *AP2/ERF* genes in the genome varied among species, ranging from 0.76% in Artichoke (*Cynara cardunculus*) to 0.43% in Medicago (*Medicago truncatula*), with an average of 0.59%—a number comparable to lettuce (Table S2). We also found a positive correlation between the number of *AP2/ERF* genes and the total gene count within each genome, with an *R*-square value of 0.68 (Table S2).

**Figure 1:**
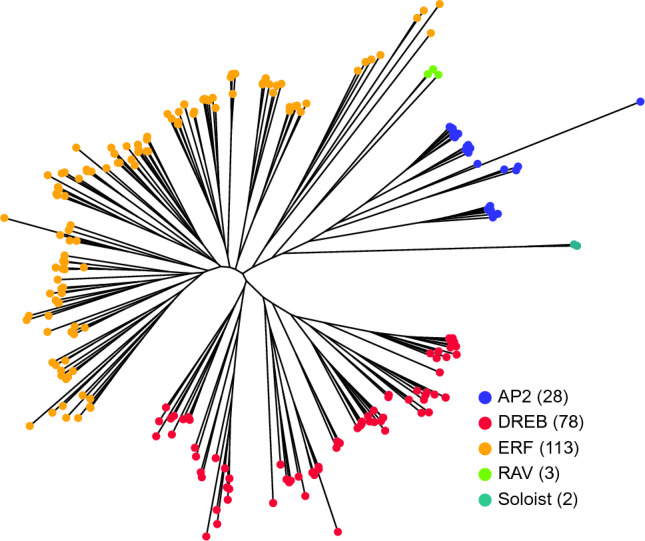
Phylogenetic tree of the *AP2/ERF* gene superfamily in lettuce constructed using the neighbor-joining method. The tree includes 224 *AP2/ERF* genes, and the subfamilies within the *AP2/ERF* superfamily are indicated by different colors. The number of members in each subfamily is provided in parentheses.

To elucidate the evolutionary relationships among the lettuce *AP2/ERF* genes, we constructed a phylogenetic tree using the neighbor-joining (NJ) method based on protein sequences. The tree revealed three main clusters: the ERF subfamily and the DREB subfamily, and a third cluster including AP2, RAV, and Soloist genes (Fig. 1). Four genes containing a single AP2 domain clustered with the AP2 subfamily genes, a pattern observed in other plant species^12,38–41^. Therefore, despite possessing a single AP2 domain, these genes were classified into the AP2 subfamily. Two genes that did not belong to any other cluster were classified as Soloists following the naming convention of Nakano et al.^12^ (Fig. 1). A few genes with a single AP2 domain were found in the third clade, but they formed a distinct subgroup separate from the AP2 subfamily genes. Thus, these genes were classified into the ERF subfamily. For clarity and ease of reference, each of the 224 genes was assigned a consecutive number: the *AP2* subfamily genes were designated as *LsAP2.1* to *LsAP2.28*; the *RAV* family genes as *LsRAV.1* to *LsRAV.3*; Soloist as *LsSoloist.1* to *LsSoloist.2*; the *DREB* family genes as *LsEFR001* to *LsERF078*; and the *ERF* family genes as *LsERF79* to *LsERF191* (Table S1).

### Phylogenetic analysis of the *DREB* and *ERF* subfamily genes

**Figure 2:**
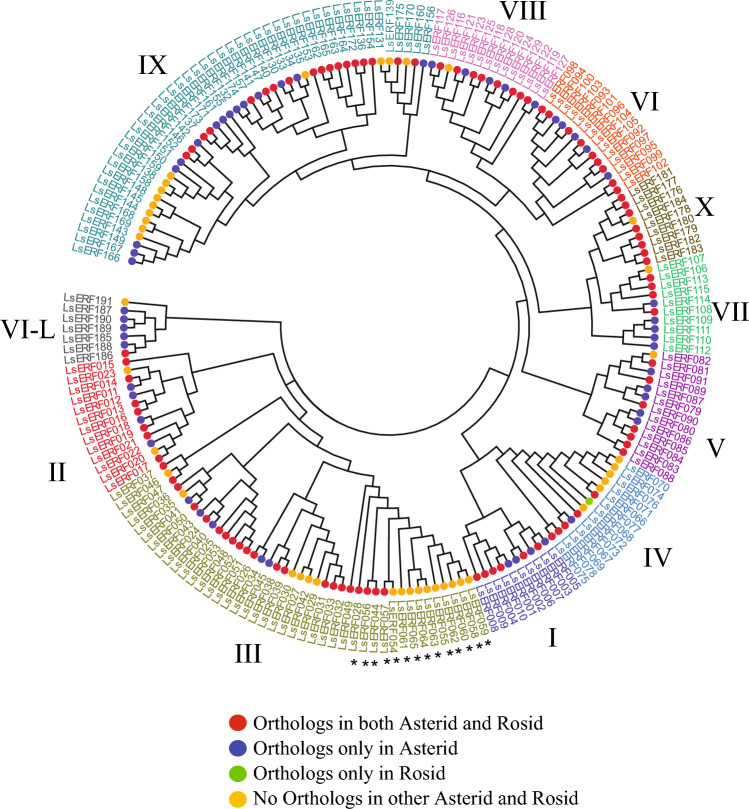
Phylogenetic analysis of 191 *ERF* genes constructed using the NJ method. The tree illustrates different subgroups, each represented by different colors. The presence of orthologs within different taxonomic groups is depicted by colored circles at the tips of the branches: red for orthologs in both the Asterid and Rosid clades; blue for orthologs in the Asterid clad only; green for orthologs in the Rosid clade only; and brown for genes with no ortholog in either clade. The fourteen *LsCBF* subfamily genes identified by Park et al.^35^ are marked by asterisks.

To determine the subgroups of the lettuce *DREB* and *ERF* subfamily genes, we adopted the classification method initially used in the previous study of *Arabidopsis* and rice. This classification involved subdividing the *ERF* and *DREB* subfamilies into several groups denoted as I–X, and VI–L^12^. We employed a combined approach of sequence similarity and phylogenetic analyses. First, we compared the lettuce protein sequences with those from *Arabidopsis* and rice using BLASTP. Based on the similarity scores from BLASTP, we assigned the lettuce genes to the same subgroup as the *Arabidopsis* or rice genes with the highest similarity. Next, we examined the assignment in the NJ tree to validate and refine the subgroup classification (Fig. 2). In general, lettuce genes classified into a particular subgroup were grouped together in the NJ tree, with a few exceptions (as shown in the Table S3). For instance, some lettuce genes, closely related to the *Arabidopsis* VIII group, fell into a cluster predominantly associated with the VI or VI–L group in the NJ tree. In such cases, we reclassified these genes as VI or VI–L based on their placement in the NJ tree. As a result, group IX appeared to be the largest with 46 genes, followed by group III with 42 genes, and group VI and VIII with 14 genes each. The smallest subfamily, VI–L, consisted of only 7 members. These subfamily sizes were comparable to those of *Arabidopsis* and rice where group IX is the largest and group III are the second largest (Table S3).

To establish orthologous and paralogous relationships of the *AP2/ERF* genes among higher plants species, we selected ten species from the Asterid and Rosid clades (Table S1). Using their genomic proteins and the orthoMCL algorithm^42^, we identified 1284 orthologous groups (Table S4). When these orthologous relationships were compared with the NJ tree, all eleven subgroups (I–X, VI–L) contained at least one gene or more orthologous to both Asterid and Rosid species, indicating that these subgroups diverged prior to the separation of the Asterid and Rosid clades (Fig. 2). The subgroups, except for group X, also contained genes only orthologous to the Asterid species, and similarly, all subgroups contained genes non-orthologous to any other species, thus specific to lettuce (Fig. 2).

Among the orthologous groups, the largest group (Group1000) contained 47 genes from the 11 species, with sunflower (*Helianthus annuus*) and *Medicago* having the highest number of genes (14 and 13, respectively) (Table S4). This group also included three lettuce genes, *LsERF028*, *LsERF056*, and *LsERF057*—also known as *LsCBF1*, *LsCBF3*, and *LsCBF4*^35^. When focusing solely on lettuce, the largest group was Group1095, containing ten lettuce genes. Notably, this group did not include any genes from other species, thus determined as lineage-specific genes. The ten paralogous lettuce genes were previously identified as the lettuce CBF subfamily, *LsCBF5*–*LsCBF14*^35^. Moreover, there were five additional groups consisting of only lettuce genes, each group containing at least two lettuce genes, resulting in a total of 25 genes (Table S4).

Overall, 54% (120) of lettuce genes had orthologs in both Asterid and Rosid species, indicating their origin from a common ancestor predating the divergence between the Asterid and Rosid clades. Additionally, 26% (58) of the genes had orthologs only in Asterid species, suggesting their evolution after divergence of Asterid and Rosid clades. Another 11% (25) had no orthologous relationship with other species, referred to as lineage-specific genes, likely evolved in the lettuce lineage (Table S4; Fig. 2). Only 0.5% had orthologs exclusively in Rosid species. The maximum orthology of lettuce *AP2/ERF* genes was observed with Artichoke (*Cynara cardunculus*, 51%), followed by Sunflower (50%), both belonging to the same family (*Asterales*) as lettuce. The least orthology was found with *Arabidopsis* (38%).

### Chromosomal distribution and gene structure of AP2/ERF proteins

**Figure 3:**
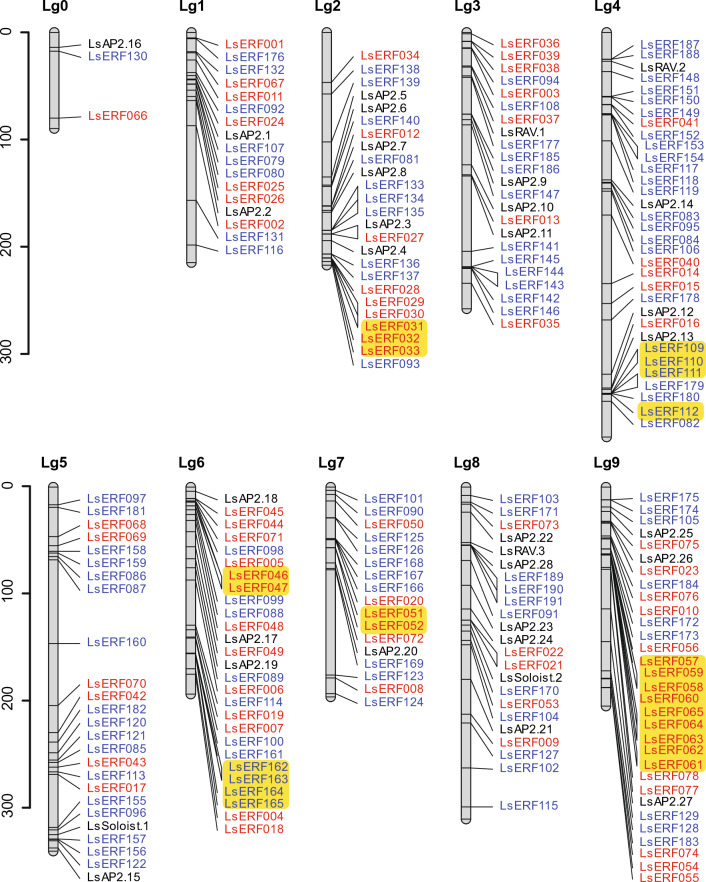
Chromosomal location of lettuce *AP2/ERF* genes on ten linkage groups. The *DREB* subfamily genes are represented in red; the *ERF* subfamily in blue; and *AP2*, *RAV*, and *Soloist* genes in black. Tandem duplicated genes are highlighted in yellow. The scale bar represents a unit of mega base pairs.

The physical locations of the *AP2/ERF* genes in the genome were relatively evenly distributed across the ten linkage groups, except for Lg0 (Fig. 3). The distribution ranged from 17 to 33 genes per linkage group (see Table S1 for precise location in the genome). However, within individual linkage groups, there were instances of uneven distribution, with several genes tandemly arrayed in close proximity.

The expansion of multigene families often involves tandem or segmental duplication^43,44^. To investigate potential tandem and segmental duplications within the lettuce *AP2/ERF* gene family, we utilized a combination of sequence similarity analysis and physical proximity within the genome. Using criteria of 80% identity and 80% coverage in pairwise BLASTP comparisons of genes, we identified 68 pairs of duplicated genes: 39 pairs were classified as tandem duplications due to their close genomic locations (Table S5), while 29 pairs were defined as segmental duplications due to their dispersed placement (Table S6). The tandemly duplicated genes fell into six clusters: two clusters on Lg6 and one cluster each on Lg2, Lg4, Lg7, and Lg9 (Fig. 3; Table S5). The largest cluster located on Lg9 comprised nine genes (*LsERF057–LsERF065,* also known as *LsCBF5–LsCBF12*). The second-largest clusters on Lg4 and Lg6 consisted of four genes each, belonging to subfamily VII and subfamily IX, respectively. The three-member cluster genes on Lg2 belonged to subfamily III. The remaining two clusters, each containing two genes, were found on Lg6 and Lg7, with genes from both clusters belonging to subfamily III.

**Figure 4:**
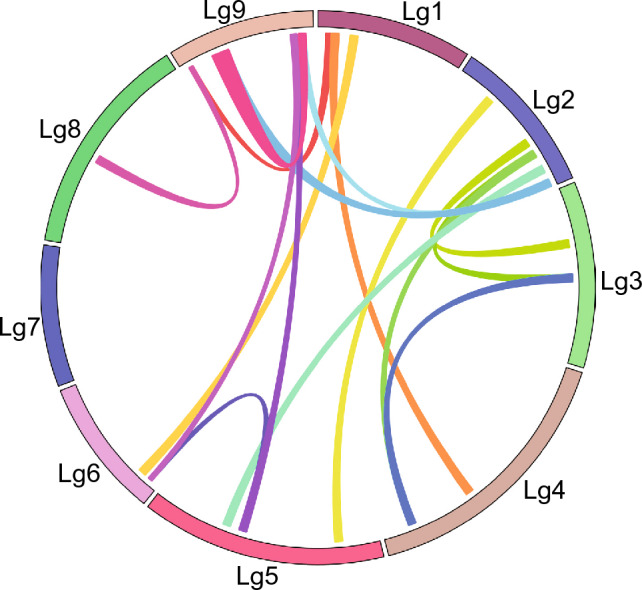
Distribution of segmentally duplicated *AP2/ERF* genes on *L. sativa* linkage groups. The duplication events are represented by colored lines, with each color signifying pairs of duplicated regions.

Regarding the segmentally duplicated gene pairs, a cluster of genes determined as tandem duplicates (*LsERF057–LsERF065* on Lg9) were also segmentally duplicated twice in the genome, resulting in three paralogs—*LsERF028* on Lg2, and *LsERF054* and *LsERF056* on Lg9 (Fig. 4; Tables S5, S6). These paralogous genes were also known as members of the *LsCBF* family, *LsCBF1*, *LsCBF13*, and *LsCBF3*. Similarly, *LsERF012* (Lg2) and *LsERF070* (Lg5) were also each segmentally duplicated twice in the genome. This duplication resulted in paralogs of *LsERF013* (Lg3) and *LsERF016* (Lg4) for *LsERF012*, and *LsERF071* (Lg6) and *LsERF074* (Lg9) for *LsERF70* (Fig. 4).

**Figure 5:**
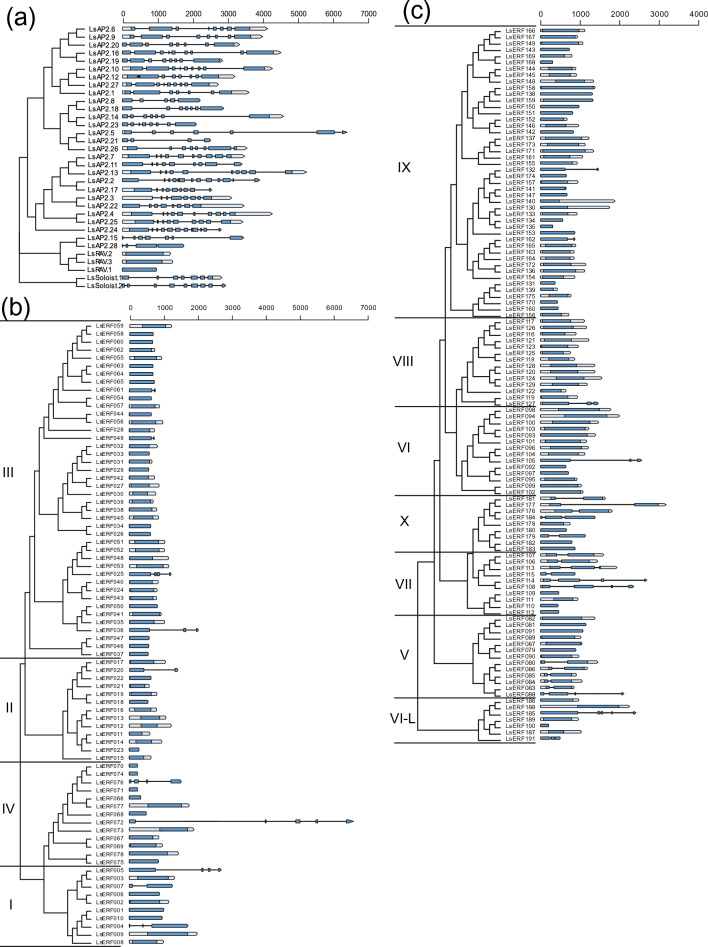
Gene structures of AP2/ERF proteins. The phylogenetic trees for the AP2 (**a**), DREB (**b**), and ERF (**c**) are constructed using the NJ method. In the illustrated gene structures, exons are depicted by blue boxes; untranslated regions (UTR) are shown in light blue; and introns are represented by black lines.

Furthermore, the exon and intron structures of the *AP2/ERF* family genes were analyzed to understand the structural diversity and its implication in the evolution of the family genes. The AP2 subfamily displayed a distinctive pattern compared to other subfamilies (Fig. 5a; Table S1). All members of the AP2 subfamily and Soloists contained introns, with the number of introns ranging from 4 to 13 per gene, whereas most members from other subfamilies were predominantly intronless. This patten has also been reported in previous studies^39–41^. Only 14% (11) of the *DREB* subfamily contained introns, ranging from 2 to 5, while 20% (23) of the *ERF* subfamily contained introns, ranging from 2 to 5 (Fig. 5b). Genes with introns in the *ERF* subfamily were largely concentrated within groups VII, V, and X, and these groups were closely placed in the NJ tree, suggesting that the gene structure has been preserved throughout the evolution of these genes (Fig. 5c).

### Divergence rate of the *AP2/ERF* genes

To understand the effect of selective constraints on the duplicated *AP2/ERF* genes, we conducted an analysis of the nonsynonymous (Ka) and synonymous (Ks) substitution ratios using the full-length protein sequences of the genes. The Ka/Ks ratio is commonly used to infer the type of selection acting on duplicated genes^45^. A high Ka/Ks ratio (more than 1) indicates that the duplicated genes have been under positive selection, possibly gaining new functions. A ratio close to 1 suggests neutral selection of the duplicated genes, with change occurring randomly without positive selection, while a low ratio (less than 1) indicates that the duplicated genes have been under purifying selection, limiting their functional divergence and maintaining their original functions.

The tandemly duplicated *AP2/ERF* gene pairs displayed a Ka/Ks ratio ranging from 0.07 to 0.57, with an average of 0.31, while the Ka/Ks ratio for segmentally duplicated gene-pairs ranged from 0.03 to 0.14, with an average of 0.20 (Table S7). In both types of duplications, the Ka/Ks ratio was significantly below 1, indicating that strong purifying selection pressure acted upon the duplicated *AP2/ERF* genes, and consequently, contributing to limiting the functional divergence of these duplicated genes.

### Expression profiling of *AP2/ERF* genes during abiotic stresses

**Figure 6:**
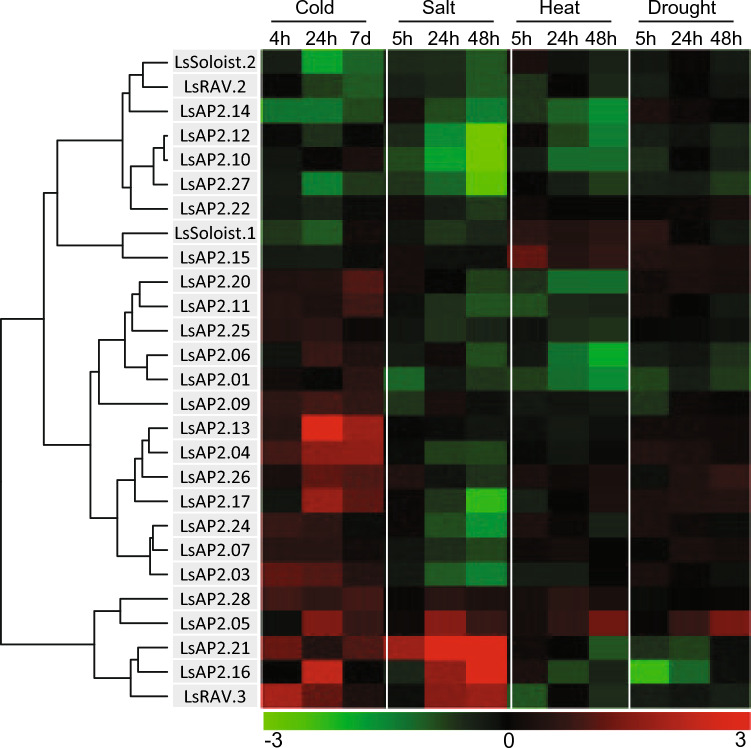
Heatmap showing the expression patterns of *AP2*, *RAV*, and *Soloist* genes in response to cold, heat, salt, and drought. Each row corresponds to a specific gene, and each column represents a stress condition. The color intensity indicates the level of gene expression (Log2 fold change): red for upregulation and green for downregulation, relative to the control condition. The heatmap was generated using the hcluster method of the R package amap^66^.

Gene expression studies provide valuable insights into the function of a gene. To investigate the roles of lettuce *AP2/ERF* genes in abiotic stresses, we analyzed the expression profiles using short-read RNA sequencing (RNA-seq) after exposing plants to various abiotic stresses: cold, heat, drought, and salt. Out of the 224 *AP2/ERF* genes, 157 genes were found to be expressed under these stress conditions (Table S8). To better illustrate the expression patterns, we constructed a hierarchical heatmap for each subfamily. In the *AP2* subfamily including *RAV* and *Soloists*, most genes were either relatively unresponsive or downregulated in response to the abiotic stresses except for cold stress (Fig. 6). This expression pattern aligns with the fact that the AP2 subfamily genes predominantly participate in the regulation of developmental processes, such as flower development, meristem determinacy, leaf cell identity, and embryo development^12^. During exposure to heat and salt, six and eight genes were significantly downregulated, respectively, while two and three genes were upregulated. In the case of drought, only one gene was significantly upregulated. Cold stress, on the other hand, led to the upregulation of six genes and downregulation of two genes. Notably, gene *LsAP2.05* showed increased expression across all four stress conditions, whereas *LsAP2.14* was consistently downregulated. *LsAP2.13* showed cold-specific upregulation with a 3.5 log2 fold change at 24 h, and *LsAP2.21* showed salt-specific upregulation with a 3.9 log2 fold change at 24 h (Fig. 6).

**Figure 7:**
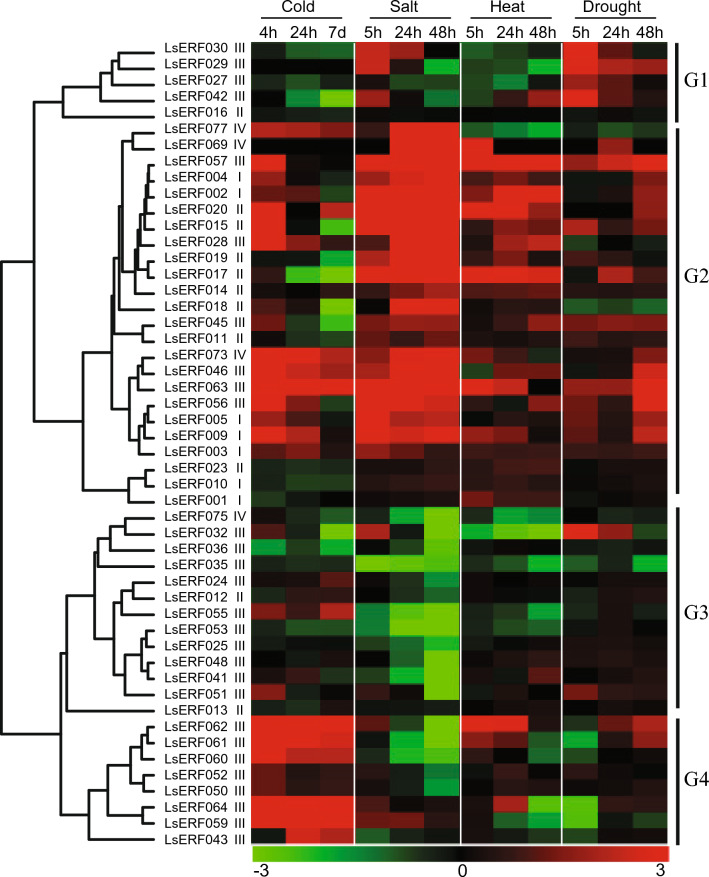
Heatmap showing the expression patterns of DREB subfamily genes in response to cold, heat, salt, and drought. Each row corresponds to a specific gene, and each column represents a stress condition. The color intensity indicates the level of gene expression (Log2 fold change): red for upregulation and green for downregulation, relative to the control condition. The heatmap was generated using the hcluster method of the R package amap^66^.

For the DREB subfamily genes, their expression patterns largely clustered into four categories: G1, primarily upregulated by salt and drought; G2, upregulated by all four stress conditions; G3, downregulated by salt or unresponsive to other stress conditions; G4, mainly upregulated by cold. The largest group, G2, suggested a role for these genes in abiotic stress signaling (Fig. 7). Within G2, three genes (*LsERF004*, *LsERF009*, and *LsERF073*) were significantly upregulated under all conditions.

Among the ten *LsCBF* genes detected as being expressed, four genes (*LsERF028*, *LsERF056*, *LsERF057*, and *LsERF063*) were placed in G2, showing upregulation by at least three stresses, while one gene (*LsERF55*) in G3 was moderately upregulated only by cold. Five genes (*LsERF059*, *LsERF060*, *LsERF061*, *LsERF062*, and *LsERF064*) in G4 were predominantly upregulated by cold. Interestingly, these five genes in G4 were identified as tandem duplicates, whereas their segmentally duplicated paralogs (*LsERF028* on Lg2 and *LsERF055* on Lg9 lower arm) were classified into groups, G2 and G3, respectively, and their tandemly duplicated paralogs (*LsERF057*, and *LsERF063*) were classified into group G2 (Fig. 7). These results implied that expression divergence occurred after duplication, potentially leading to functional divergence.

**Figure 8:**
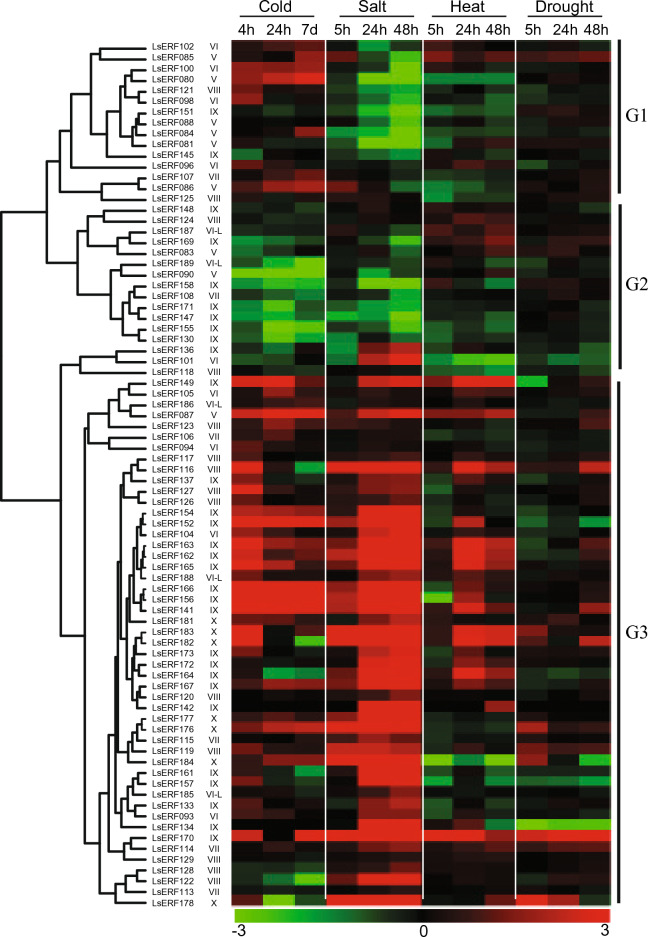
Heatmap showing the expression patterns of ERF subfamily genes in response to cold, heat, salt, and drought. Each row corresponds to a specific gene, and each column represents a stress condition. The color intensity indicates the level of gene expression (Log2-fold change): red for upregulation and green for downregulation, relative to the control condition. The heatmap was generated using the hcluster method of the R package amap^66^.

The ERF subfamily genes exhibited three main expression patterns: G1, slightly upregulated by cold alone; G2, mostly downregulated by all stress conditions; and G3, generally upregulated by all stress conditions, with salt causing the most significant increase (Fig. 8). Remarkably, two genes (*LsERF085* and *LsERF116*) displayed upregulation across all four stress conditions. Ten genes were activated exclusively by cold stress, five by salt stress, and three exclusively by heat stress. The observed diversity in gene expression patterns suggests a critical role of the *AP2/ERF* genes in modulating complex stress response pathways, ultimately facilitating stress adaptation and multi-stress tolerance in lettuce.

## Discussion

The AP2/ERF superfamily is recognized across various plant species as pivotal transcription factors in abiotic stresses^12,38,39,41,46,47^. Despite its importance, a comprehensive understanding of this family in lettuce has remained elusive. In this study, we undertook a genome-wide search for *AP2/ERF* family genes in lettuce and identified 224 *AP2/ERF* genes, which account for 0.59% of the total coding genes in the genome (Table S2). This percentage varies across plant species, ranging from 0.77% in Artichoke to 0.43% in Medicago. This variation can be partly attributed to the gene duplication events that have occurred during the evolutionary development of this family. We analyzed the duplication events based on sequence similarity and physical distance within chromosomes. Our analysis identified 39 pairs of tandem duplication and 29 pairs of segmental duplication, together accounting for 21% (48) of the AP2/ERF family (Tables S5 and Table S6). This finding illustrates that gene duplication has played a critical role in expanding the lettuce AP2/ERF, a pattern also evident in diverse plant species^38–40^. Interestingly, some of the tandem duplications were also found to be segmentally duplicated. For instance, a cluster of genes (*LsERF057*–*LsERF065*, also referred to as *LsCBF4*–*LsCBF12*) located on Lg9 was segmentally duplicated twice, giving rise to genes *LsERF054* and *LsERF055* on the lower arm of Lg9, and *LsERF028* on Lg2 (Fig. 4; Table S6). These paralogous genes, as members of the *LsCBF* subfamily, are well-known for their important roles in cold signaling pathway. Through orthology analysis, we further explored the evolutionary relationships of these duplicated genes. The clusters of *LsERF057*–*LsERF065* on the upper arm of Lg9 except for *LsERF057*, and *LsERF054* and *LsERF055* on the lower arm of Lg9, were identified as lettuce lineage-specific genes (Table S4), while the *LsERF028* (Lg2) and *LsERF057* (Lg9) were orthologous with genes from both Asterid and Rosid species. These orthologous relationships suggest that *LsERF028* (Lg2) and *LsERF057* (Lg9) genes are more ancient, originating from a shared ancestor of the Asterid and Rosid clades. Subsequently, these genes were duplicated either segmentally or tandemly, resulting in the cluster of *LsERF058*–*LsERF065* on the upper arm of Lg9, and *LsERF054* and *LsERF055* on the lower arm of Lg9.

Duplication is a well-recognized mechanism contributing to genetic variation, often leading to subfunctionalization or neofunctionalization of genes^48^. When functional redundancy arises from gene duplication, the subsequent accumulation of mutations can promote divergence and expansion within the gene family^49,50^. Despite this potential for divergence, duplicate genes can often be preserved through selective constraints such as purifying selection. This preservation is likely driven by the genes’ important roles in crucial biological processes like abiotic stresses, where the purifying selection eliminates deleterious mutations to maintain the ancestral function of the duplicates. Our analysis of the Ka/Ks ratio supports the prevalence of purifying selection among the lettuce *AP2/ERF* gene family. The Ka/Ks ratios between pairs of duplicated genes ranged from 0.038 to 0.57, figures significantly lower than 1 (Table S7). These ratios are indicative of strong purifying selection pressure, constraining the divergence of the duplicated AP2/ERF proteins to preserve their functions. This purifying selection may confer advantages against abiotic stresses, exemplified by the gene dosage effect where increased production of gene products can lead to a rapid and robust response to sudden environmental changes. For example, *Arabidopsis CBF* genes demonstrate this dosage effect in response to cold stress. When individual *CBF* genes are mutated in *Arabidopsis*, the freezing tolerance of plants is impaired in direct proportion to the number of mutated genes, indicating that CBF proteins function additively to bolster freezing tolerance^8,51^.

While selective constraints on protein sequence may limit the functional divergences of duplicated genes, the proteins could acquire novel functions through altered gene expression. For instance, modifications of promoter regions can lead to different spatial or temporal expression patterns, resulting in functional divergence. In *Arabidopsis*, the proteins DREB2 (VI subfamily) and CBF/DERB1(III subfamily) share high sequence similarity and regulate a similar set of downstream target genes, as both family genes can bind to DRE/CRT cis-elements^14,15^. However, they are involved in different abiotic stress responses: the *CBF/DERB1* genes primarily respond to cold signaling, while the *DREB2* genes predominantly respond to drought signaling^14,15^. This distinction in stress response is due to differentiation in their promoter regions, resulting in different responsiveness to stresses. We observed a similar phenomenon in the duplicated genes within the lettuce AP2/ERF family (Fig. S1). Members of duplicated genes displayed divergent expression patterns, revealing that some of the duplicated genes have undergone functional divergence through altered expression, possibly driven by promoter differentiation. Specifically, the ancient genes *LsERF028* and *LsERF057,* originating from a common ancestor of Asterid and Rosid clade—also known as *LsCBF1* and *LsCBF4*, respectively^35^—displayed strong activation in response to salt stress (Fig S1). In contrast, most of their segmentally or tandemly duplicated paralogs were activated predominantly by cold stress. These expression patterns are consistent with the qPCR results of a previous study^35^, indicating that the later duplicated genes acquired altered expression, contributing to functional divergence among the duplicated genes. Our findings underscore the importance of purifying selection in maintaining the lettuce AP2/ERF gene family, while also suggesting that promoter differentiation may play a role in functional divergence within the family, ultimately contributing to the adaptation of lettuce to various abiotic stresses.

The orthology analysis indicates that most *AP2/ERF* family genes (88%) are orthologous to genes from either Asterid, Rosid, or both species, while around 12% of genes do not have any ortholog among the ten Asterid and Rosid species, suggesting that these genes are specific to the lettuce lineage (Table S4). These lineage-specific genes may have evolved during lettuce speciation, possibly playing important roles in adapting to the conditions that lettuce species faced during evolution. Among the lineage-specific genes, the largest group consists of ten paralogous *LsCBF* genes (Group1095) that were generated through tandem or segmental duplications. CBF transcription factor genes are known for their important roles in cold stress adaptation^18^. The finding that a group of *LsCBF* genes are lettuce-lineage specific, suggests that the expansion of the CBF family in lettuce might occur during its speciation, perhaps to adapt to cold stress conditions that lettuce encountered during its evolution. The findings align with a previous study by Park et al.^35^, where they observed that CBF genes from diverse species including lettuce were distinctly grouped by species in a phylogenetic tree. Moreover, these *CBF* genes were found in tandem on the genome within each plant species. Such clustering in the NJ tree and physical proximity on the chromosomes suggested that paralogous tandem duplications of the *CBF* genes occurred in each species lineage. Our orthology and duplication analyses provided strong evidence supporting the notion that, at least in lettuce, CBF genes evolved through both tandem and segmental duplications in the lettuce lineage. The expansion of the CBF subfamily in lettuce potentially serve as an adaptation strategy to cope with cold stresses that might be prevalent during lettuce lineage evolution.

Some angiosperm families evolved the ability to adapt to cold temperatures during the global cooling climate, extending from the mid-Eocene (46 million years ago) to the late Oligocene (27 million years ago)^52^, resulting in their expansion into temperate regions. In a recent study by Zhang et al.^53^, molecular evolution analysis demonstrated a dramatic increase in the copy number of *CBF* genes in the Pooideae family (which includes 3900 species including wheat and barley) through tandem duplication during the Eocene–Oligocene transition. They suggested that this duplication likely facilitated the successful adaptation of Pooideae members to temperate regions by fostering resilience to cold habitats, highlighting the importance of genetic innovation in plant adaptation to local environmental conditions. Understanding the molecular basis of this gene family expansion and functional diversification in lettuce can provide valuable insights into the plant ability to thrive in various environmental challenges, ultimately contributing to the improvement of lettuce crop production under adverse conditions.

The RNA expression signals of *AP2/ERF* genes in lettuce, when exposed to various stress conditions, illuminate their potential roles in abiotic stress responses. Among the 224 genes, approximately 47% (105 genes) showed significant induction in response to at least one of the examined stress conditions. Interestingly, some genes were found to be responsive exclusively to a particular stress stimulus (Figs. 6, 7, 8). For example, 25 genes (5 from *AP2*, 10 from *DREB*, 10 from *ERF*) exhibited specific upregulation in response to cold stress at one or more time points. Similarly, 24 genes (2 from *AP2*, 6 from *DREB*, 16 from *ERF*) were selectively responsive to salt stress, while five genes (1 from *AP2*, 1 from *DREB*, 3 from *ERF*) responded specifically to heat stress, and two genes (exclusively from *DREB*) showed upregulation in response to drought stress (Table S8). These stimulus-specific genes hint at a fine-tuned regulation of response mechanisms to particular environmental cues. Considering that plants often face multiple stress conditions simultaneously, leading to more severe damage, the six genes (*LsAP2.05*, *LsERF004*, *LsERF009*, *LsERF073*, *LsERF085*, *LsERF116*) that showed significant upregulation across all four stresses are of particular interest. These genes may serve as potential candidate genes for further functional validation and utilization in crop improvement programs aimed at comprehensive stress resistance. The universal or stimulus-specific expression patterns in the *AP2/ERF* gene family expanded our understanding of the molecular basis of stress tolerance and adaptation in plants.

In conclusion, our study significantly contributes to our understanding of the evolutionary dynamics of the AP2/ERF transcription factor family in lettuce. By uncovering the genetic basis of stress responses, our findings lay a strong foundation for future studies on stress tolerance and adaptation mechanisms in lettuce.

## Methods and materials

### Plant material and growth conditions

Plants were grown in soil pots in growth chambers, where temperature was maintained at 20 °C and a photoperiod of 16 h of light and 8 h of darkness was applied. The light intensity ranged from 350 to 400 μmol m^−2^ s^−1^. Abiotic stress treatments were conducted on eighteen-day-old plants, as described in Park et al.^35^. For cold stress treatment, plants were exposed to 4 °C for 4 h, 24 h, or 7 days with a light intensity of 100 μmol m^−2^ s^−1^. The other stress treatments were carried out for 0 h, 5 h, 24 h, and 48 h with a light intensity of 300 μmol m^−2^ s^−1^. For high salt stress conditions, plants were treated with 250 mM NaCl^54^. For heat stress conditions, plants were exposed to 34 °C^55^. For drought stress conditions, watering was withheld after ensuring all excess water was drained and absorbed by paper towels from the pots. Following exposure to these stress conditions, leaf samples were collected for each treatment, with the 0 h samples serving as controls. All procedures were conducted in accordance to the guidelines of USDA-ARS.

### Sequence retrieval and identification of AP2/ERF proteins from *L. sativa*

The lettuce protein database (genome version 8, id37106) was obtained from the CoGe genome evolution platform (https://genomevolution.org/coge). In cases where there were multiple isoforms for a gene in the protein database, the protein which has the highest amino acid sequences was selected as a representative for the gene. The Hidden Markov Model profiles of the AP2/ERF domain (PF00847) and B3 domain (PF02362) were obtained from the Pfam v27.0 database (http://Pfam.sanger.ac.uk/). To identify AP2/ERF proteins in lettuce*,* the AP2 domain profile was searched against the lettuce protein data using the hmmsearch tool implemented in HMMER3 v3.2.1 (http://hmmer.org). The proteins with an AP2 domain match E-value of 1e − 5 or lower were selected for further analysis. The final non-redundant AP2/ERF protein sequences were confirmed for the presence of AP2/ERF domain using the HMMSCAN (http://hmmer.janelia.org/search/hmmscan). For RAV subfamily, the B3 domain was searched against all AP2/ERF proteins using the hmmsearch function. Hits with an E-value of lower than 1e-5 were designated as members of the RAV subfamily.

### Gene nomenclature

The naming convention of gene models in this study was modified from the annotation of the lettuce genome v8. Each gene name follows a specific format, which includes the following components: (1) The prefix 'Ls' indicating the lettuce species, abbreviated from *L. sativa*; (2) A one digit number indicating the linkage group (0–9); (3) The letter, 'g' indicating that the name is assigned for a gene; and (4) A 4–6 digit number unique to each gene, assigned from the lettuce genome v8. For example, a gene name in the genome v8, such as ‘Lsat_1_v5_gn_4_156100.1’ can be simplified to ‘Ls4g156100.1’.

### Phylogenetic analysis

Phylogenetic trees were constructed based on protein sequences. Initially, multiple protein sequences were aligned using MUSCLE5^56^ with the default parameter setting. The resulting alignment was then manually inspected and adjusted, if necessary, using BioEdit^57^. The phylogenetic tree was generated based on the aligned sequences using the neighbor-joining method in MEGA version 11^58^ with the parameters of p-distance model, uniform rates among sites, and partial deletion of sites with less than 95% data. The resulting trees were visualized using FigTree version 1.4.4 (http://tree.bio.ed.ac.uk/software/figtree). To assign subgroups in the lettuce AP2/ERF family, AP2 family genes from *Arabidopsis* thaliana (At) and *Oryza sativa* (Os) were obtained from the Plant transcription factor database (http://plntfdb.bio.uni-potsdam.de/v3.0/). The *AP2/ERF* protein sequences were then subjected to BLASTP against *Arabidopsis* and rice protein sequences. Following the methods described in Nakano et al.^12^, the genes were assigned to specific subgroups. In cases where the subgroup assignments between *Arabidopsis* and rice did not agree, the assignment followed that of *Arabidopsis*.

### Chromosomal location and gene structural analysis

The genomic coordinates of the *AP2/ERF* genes in lettuce were obtained from the genome annotation information. The genes were then mapped onto the ten lettuce chromosomal linkage groups based on their physical positions in base pairs (bp). The location of the genes on the physical map of each chromosome were visualized using the R package LinkageMapView^59^.

For the gene structure analysis including exon, intron, and UTR regions, the structural information of genes was obtained from the *L. sativa* genome database (version 8). Diagrams illustrating the exon–intron architecture of the genes were constructed using a custom R script.

### Duplication analysis and Ka/Ks ratio estimation

To assess the contribution of segmental and tandem gene duplications to the genome-wide expansion of the AP2/ERF family in lettuce, genes located within 5-Mb regions and exhibiting 80% or higher similarity with > 80% coverage on both query and hit genes were considered as tandemly duplicated genes. On the other hand, genes satisfying the same criteria but separated by greater than 5 Mb were identified as segmentally duplicated genes.

To estimate the selective pressure acting on duplicated genes, synonymous (Ks) and non-synonymous (Ka) substitutions per site between the duplicated genes were calculated. Protein sequences of each pair of duplicated genes were globally aligned using CLUSTALW2^60^, and the protein sequence alignments were converted to DNA alignments based on their corresponding DNA sequences. From these DNA alignments, the Ka, Ks, and the significance of Ka/Ks were computed using ‘KaKs_calculator’ version 1.2 with a model-average method^61^. The significance of Ka/Ks, which indicates whether the duplicated genes have undergone positive selection or purifying selection, was tested using the Fisher’s exact test. The ratios with a P-value of at least 0.01 were considered statistically significant.

### Determination of orthologous relationships

To determine orthologous proteins among higher plant species for the lettuce *AP2/ERF* genes, five plant species were selected from each of the Asterid and Rosid clade. Genomic protein databases for the ten species were obtained from NCBI database. The genomic proteins were screened for AP2-domain containing proteins using HMMER3 v3.2.1 (http://hmmer.org), with the AP2 domain profile (Pfam accession, PF00847) as a query. Proteins with truncated AP2 domains or AP2 domain match E-values exceeding 1e − 5 were excluded from subsequent analyses.

For the assignment of proteins into orthologous clusters, the OrthoMCL program was employed^42^. To ensure data quality, low-quality protein sequences with length less than 30 amino acids were removed, and the remaining protein sequences were modified following the OrthoMCL requirements, including appending a species-specific prefix to each protein name. Next, an all-versus-all BLASTP search was performed with an E-value cutoff of less than 1e − 5 to establish pairwise similarities between proteins, including lettuce AP2/ERF proteins. The BLASTP results were parsed by the OrthomclBlastParser function and loaded into a local SQLite orthoMCL database. Potential pairs of proteins that represented orthologs, in-paralogs, or co-orthologs were identified using the OrthoMCL algorithm among the proteins. Subsequently, the potential pairs were classified into orthologous clusters using the MCL algorithm^62^ with an inflation parameter of 2.

### RNA sequencing analysis

Leaf tissues were harvested from cultivar ‘Salinas’ plants exposed to salt, heat, and drought for 0 h, 5 h, 24 h and 48 h with triplicated biological controls. To mitigate systemic bias among samples, the plants were grown using a randomized block design and were rotated periodically. For each biological replicate, total RNA was isolated using Plant RNeasy kit (Qiagen) and submitted to Novogene Corporation (https://en.novogene.com/) for RNA sequencing. The sequencing was conducted on the Illumina HiSeq platform, generating 150 bp paired-end reads (http://www.illumina.com). The RNA-seq data of cold treatment (GSE134012) was obtained from Gene Expression Omnibus (www.ncbi.nlm.nih.gov/geo/). The RNA-seq reads were aligned to the *L. sativa* reference genome (version 8) using STAR version 2.5.2^63^. The resulting alignments (BAM files) were used to count reads at the gene level by using featureCounts program^64^. Differentially expressed genes were determined by using the R package edgeR, version 3.5.0^65^.

To account for potential inflation in differential gene expression estimates due to low expression, only genes with a minimum of 0.5 read per million (< 0.5 CPM) in at least two samples were included. Genes exhibiting a two-fold change (log2 = 1) or more and an FDR = 0.01 were determined as differentially expressed.

To examine RNA expression patterns across the stress conditions, hierarchical clustering analyses were conducted using the hcluster method of the R package amap (version 0.8.16)^66^, and the resulting clusters were visualized using the heatmap.2 method of the R package gplots version 3.1.3^67^.

### Supplementary Information

Supplementary Information.

Supplementary Tables.

## Data Availability

The RNA-seq data are available in the Gene Expression Omnibus (www.ncbi.nlm.nih.gov/geo/) under accession number GSE241604. All relevant data are included in the manuscript and the Supporting Information files.
