# Supplementary material for: Genome-wide characterization and evolutionary analysis of the AP2/ERF gene family in lettuce (Lactuca sativa)
Source: Sci Rep. 2023 Dec 11;13:21990. doi: 10.1038/s41598-023-49245-4 (PMC10713603; doi:10.1038/s41598-023-49245-4)
Supplement: Supplementary file 1 — Supplementary Information. [file 41598_2023_49245_MOESM1_ESM.docx]

**Genome-wide characterization and evolutionary analysis of the *AP2/ERF* gene family in lettuce (*Lactuca sativa*)**

**Sunchung Park^1,*^, Ainong Shi^2^, Lyndel W. Meinhardt^1^, and Beiquan Mou^3^**

^1^U.S. Department of Agriculture, Agricultural Research Service, Beltsville, MD 20705, USA

^2^Horticulture Dept., University of Arkansas, Fayetteville, AR 72701, USA

^3^U.S. Department of Agriculture, Agricultural Research Service, Salinas, CA 93905, USA

**Supplementary information**

Supplementary Figure S1

Supplementary Table S1-6

**
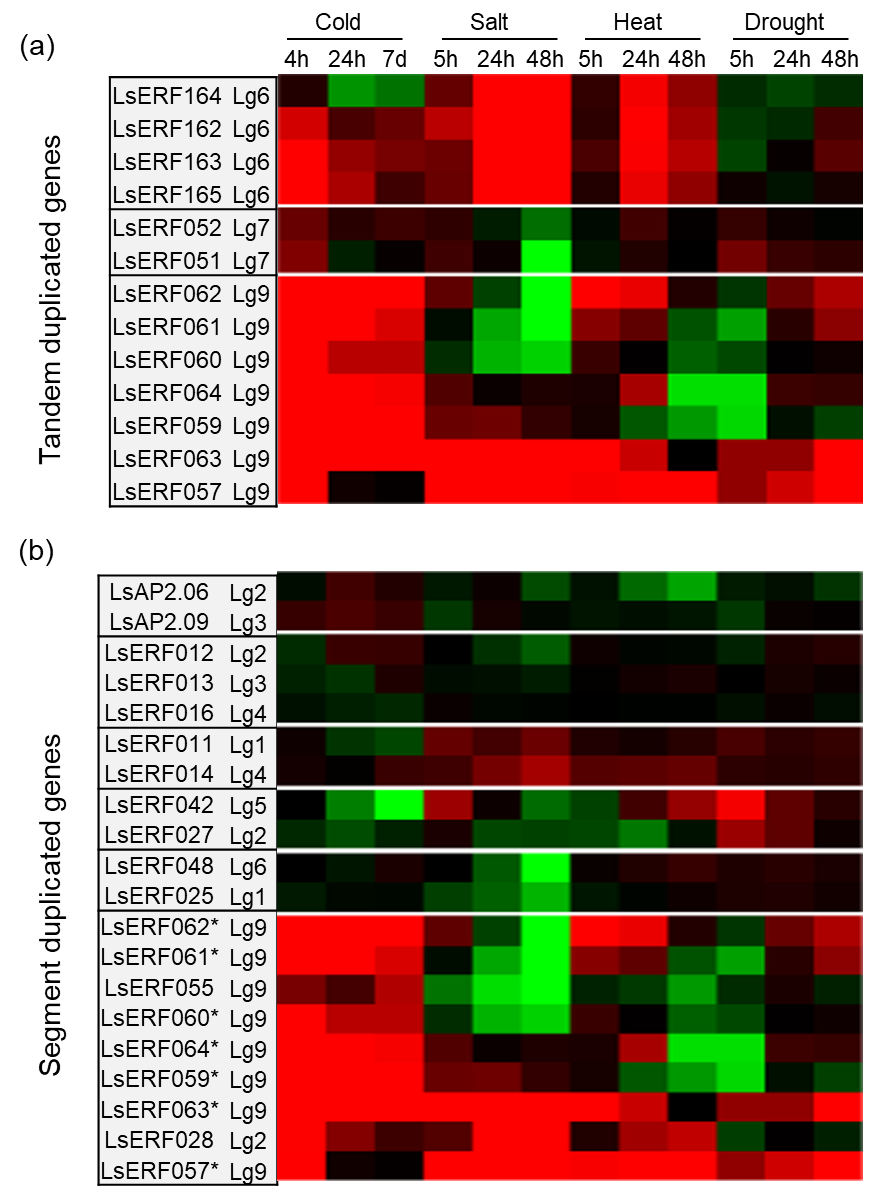
**

**Figure S1. The heatmap showing the expression patterns of tandemly (a) and segmentally (b) duplicated genes in response to cold, heat, salt, and drought.** Each row corresponds to a specific gene, and each column represents a stress condition treated for hours (h) or days (d). The color intensity indicates the level of gene expression: red for upregulation and green for downregulation, relative to the control condition. The asterisks indicate the genes that were also determined as tandem duplicates, and those tandem duplicates were segmentally duplicated to *LsERF028* and *LsERF55* (Table S5; Table S6).

**Supplementary Tables**

**Table S1. List of all 224 AP2/ERF genes in lettuce**

**Table S2. List of plant species from Asterid or Rosid subclasses used in this study**

**Table S3. BLASTP results of Lettuce AP2/ERF genes against Arabidopsis and Rice**

**Table S4. List of orthologous groups among ten plant species**

**Table S5. List of a pair of genes determined as tandem duplicates**

**Table S6. List of a pair of genes determined as segmental duplicates**

**Table S7. Ka/Ks ratios for pairwise comparisons among the duplicated AP2/ERF genes**

**Table S8. Expression signals and false discovery rate (q-value) of AP2/ERF genes**
